# Nanosurfaces modulate the mechanism of peri-implant endosseous healing by regulating neovascular morphogenesis

**DOI:** 10.1038/s42003-018-0074-y

**Published:** 2018-06-18

**Authors:** Niloufar Khosravi, Azusa Maeda, Ralph S. DaCosta, John E. Davies

**Affiliations:** 1 Institute for Biomaterials and Biomedical Engineering, University of Toronto, Toronto, ON M5G 1G6 Canada; 2 Faculty of Dentistry, University of Toronto, Toronto, ON M5G 1G6 Canada; 3 Princess Margaret Cancer Center, University Health Network, Toronto, ON M5G 1L7 Canada; 4 Department of Medical Biophysics, University of Toronto, Toronto, ON M5G 1L7 Canada; 5 Techna Institute, University Health Network, Toronto, ON M5G 1L5 Canada

## Abstract

Nanosurfaces have improved clinical osseointegration by increasing bone/implant contact. Neovascularization is considered an essential prerequisite to osteogenesis, but no previous reports to our knowledge have examined the effect of surface topography on the spatio-temporal pattern of neovascularization during peri-implant healing. We have developed a cranial window model to study peri-implant healing intravitally over clinically relevant time scales as a function of implant topography. Quantitative intravital confocal imaging reveals that changing the topography (but not chemical composition) of an implant profoundly affects the pattern of peri-implant neovascularization. New vessels develop proximal to the implant and the vascular network matures sooner in the presence of an implant nanosurface. Accelerated angiogenesis can lead to earlier osseointegration through the delivery of osteogenic precursors to, and direct formation of bone on, the implant surface. This study highlights a critical aspect of peri-implant healing, but also informs the biological rationale for the surface design of putative endosseous implant materials.

## Abstract (web summary)

Niloufar Khosravi et al. present a cranial window model to facilitate the study of neovascularization during healing around metallic implants. They find that the topography of the implant can be engineered to improve the formation of bone over the implant through an expedited neovascularization.

## Introduction

Each year millions of metallic surgical implants are placed in patients worldwide, which include total hip replacements, dental implants and knee prostheses, screws to secure spinal fixation devices and anchorage components for facial prostheses, hearing aids, and orthodontic appliances. With the exception of cemented prostheses, osseointegration is crucial to the functional success of such endosseous devices. Osseointegration may be achieved by either contact or distance osteogenesis—the formation of bone directly on the implant surface, or the old bone surface, respectively. While initial implant stability may be achieved by physical engagement in cortical bone, contact osteogenesis will only occur through bone remodeling. On the contrary, in the trabecular bony compartment, contact osteogenesis can provide rapid bony anchorage due to the recruitment and migration of osteogenic cells (osteoconduction) from the marrow interstices to the implant surface^1^.

It is generally accepted that the mesenchymal progenitors of osteogenic cells are perivascular cells^2,3^, although little is known about how and when these cells enter the wound site. Neovascularization, or formation of new blood vessels, is a critical prelude to osteogenesis. Neovascularization may occur through either angiogenesis and/or vasculogenesis^4,5^; and it can be assumed that the incursion of perivascular cells is dependent upon neovascularization. Neovascularization may occur through a variety of mechanisms^6–11^ that lead, through maturation, to the establishment of a hierarchical functional vascular network. While implant surface design is considered a critical driver of osteoconduction, and topographically complex implants have been shown to increase bone implant contact (BIC)^12–14^, no evidence has emerged to suggest that implant topography has an influence on peri-implant neovascularization.

It has been shown that implant surfaces increase platelet and neutrophil adhesion and activation^15–17^ that lead to an increased level of local angiogenic and osteogenic growth factors and cytokines^18^. Furthermore, micron-scale roughness on titanium (Ti) implants has been shown to stimulate the secretion of pro-inflammatory cytokines by macrophages including tumor necrosis factor-α^19^, which primes endothelial cells for angiogenic sprouting^20^. Indeed, some authors have reported that rough implant surfaces affect endothelial cell proliferation, motility^21^, and endothelialization (tube formation)^22^. To complement these in vitro reports, upregulation of angiogenic and osteogenic genes has been reported following clinical insertion of topographically complex Ti implants^23^.

Thus, the available evidence would suggest that implant surface topography has profound effects on the cellular mechanisms of peri-implant healing. Indeed, due to the importance of the wound neovascularization, biomedical materials have been developed for local delivery of an angiogenic gene^24^. However, the possible effects of implant surface topography on the phenomenon of peri-implant neovascularization in vivo remain unexplored.

To address this issue, we have developed a new in vivo experimental murine model to track the spatiotemporal development of neovascularization in the peri-implant healing compartment as a function of implant surface topography. The model integrates a custom-designed cranial metallic implant with an optically transparent window chamber that is compatible with both confocal-based and multiphoton-based intravital microscopic imaging systems.

We first use the model to demonstrate the expected outcomes of contact and distance osteogenesis on nanotopographically complex (TiNT) and machined-surfac (TiMA) Ti implants, respectively, as they would be expected to exhibit contact and distance osteogenesis^14,25^. Then, we demonstrate that differences in the topography of the surface are reflected in significantly different patterns of peri-implant neovascularization. Quantitative intravital confocal imaging reveals that changing the implant surface topography has a substantial effect on peri-implant neovascular morphogenesis. Specifically, new blood vessels develop proximal to, and fully functional vasculature matures sooner in the presence of, a nanotopographically complex implant surface.

## Results

### Cranial implant window chamber (CIWC) model

**Fig. 1:**
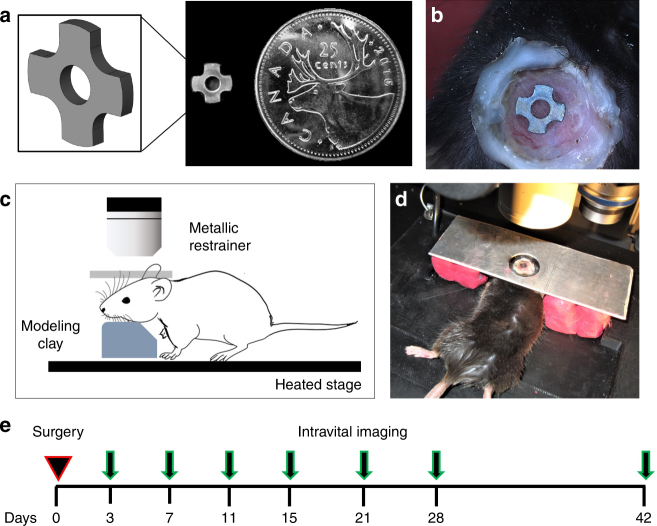
The cranial implant window chamber (CIWC) developed to study peri-implant vascularization and osteogenesis intravitally and longitudinally. **a** The cruciate Ti implant (4 mm in diameter) provided four distinct healing volumes that were selected as ROIs to examine microvascular growth and bone healing dynamics (*Note*: The central hole was designed to aid implant production and handling, as described in the text). **b** Photograph of the CIWC surgically implanted in the calvaria of a living mouse. The cover-slip window was fixed peripherally with dental restorative material (white), stabilizing the implant in the defect. **c**, **d** A schematic demonstrating a mouse on the heated microscope stage. The head was immobilized by a custom-made metallic restrainer above and modeling clay below to minimize motion artifacts during intravital optical imaging. **e** The experimental timeline from the day of the surgery showing the subsequent imaging time points

The CIWC was designed to fit precisely into a trephined calvarial defect of 4.0 mm diameter (Fig. 1a). The friction fit between the periphery of the implant and the marginal bone provides the initial stability of the implant. The four cut-outs, making the cruciate shape, provide four distinct healing volumes (regions of interest) to examine neovascularization and bone formation over time (Fig. 1b–e), which are two crucial steps to integrate the Ti implant in the calvarial bone.

**Fig. 2:**
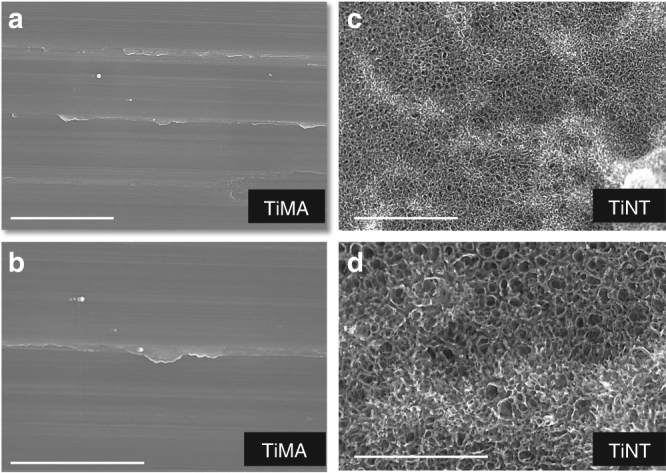
Imaging of the topography of the unmodified and modified surfaces by FE-SEM. Photomicrographs of the Ti implant surfaces at 20 and 50k magnifications. **a**, **b** TiMA surface. **c**, **d** Grit blasted/acid etched and nanotube (TiNT) surface. Scale bar 2 µm (**a**, **c**), 1 µm (**b**, **d**)

The surface topographies of both TiMA and TiNT implants were characterized by field emission scanning electron microscopy (FE-SEM). At lower magnifications machining marks were still visible on the TiMA implants (Fig. 2a), but at higher magnifications they were essentially devoid of surface features (Fig. 2b). On the contrary, TiNT surfaces showed both the micron-scale topography created by grit blasting and acid etching (Fig. 2c), and a superimposed nanotopography due to the creation of nanotubes (Fig. 2d).

**Fig. 3:**
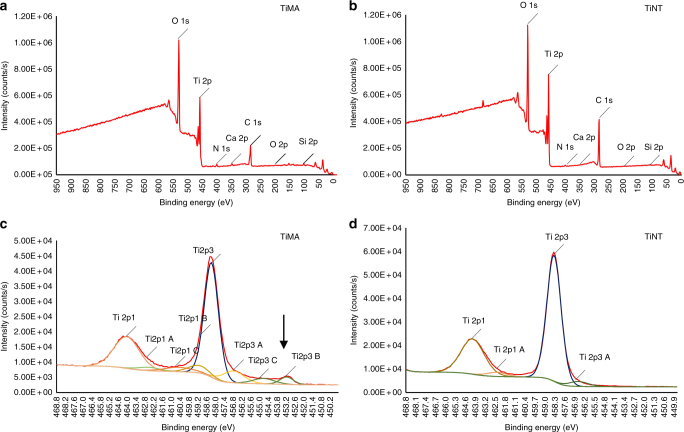
XPS of the implant surfaces. Survey spectra of **a** the TiMA and **b** TiNT surfaces. The two spectra appeared very similar in elemental composition indicating no notable surface contamination due to the multiple processing steps required for the TiNT surface. **c**, **d** Deconvolution of the Ti envelop for **c** TiMA surface and **d** TiNT surface. TiMA surface had a thin (<10 nm) TiO_2_ layer as the underlying metal peak is seen at 453.2 eV (arrow). On the contrary, this and the sub-oxide peaks at <456 eV were absent in the TiNT surface, on which the oxide layer was >10 nm (the sampling depth of the technique)

To test whether the complex grit blasting, acid etching, and nano-tube creation on the TiNT surfaces induced chemical differences between TiMA and TiNT surfaces, we analyzed the elemental composition of the lateral and top surfaces of each type of Ti implant by X-ray photoelectron spectroscopy (XPS). Survey spectra of TiMA and TiNT samples are shown in Fig. 3a and b, and Table 1 lists the relative atomic percentage for each of the elements labeled in Fig. 3a and b. The same three predominant peaks, O1s, Ti2p, and C1s are visible in the survey spectra for TiMA and TiNT surfaces with no discernable distinctions in the minor peaks—N 1s, Ca 2p, and Si 2p. Since C1s is from adventitious carbon the only two relevant elements are Ti and O. Since the O1s envelope will have contributions from C–O groups (note the increase in O1s in the TiMA group is inversely proportional to the decrease in C1s, with respect to the TiNT group), we have only focused on the Ti envelop in our deconvolution.

**Table 1:** Elementary composition (relative atomic percentage) of machined (TiMA) and nanosurface (TiNT)

	Si2p	Cl2p	C1s	Ca2p	N1s	Ti2p	O1s	Zn2p3
*Elemental composition (atomic %)*								
TiMA-top-1	0.98	0.28	27.13	0.3	1.46	21.28	47.5	1.08
TiMA-top-2	1.95	0.23	24.81	0.34	1.44	21.77	49.1	0.37
TiMA-side-1	0.87	0.28	40.54	0.25	1.39	15.66	39.04	1.97
TiMA-side-2	2.54	0.24	30.41	0.44	1.68	18.31	46.09	0.28
Average	1.585	0.2575	30.7225	0.3325	1.4925	19.255	45.4325	0.925
TiNT-top-1	0.27	0.08	38.75	0.04	0.8	20.03	39.95	0.07
TiNT-top-2	0.19	0.1	42.22	0.04	0.9	18.53	37.92	0.09
TiNT-side-1	0.27	0.08	38.75	0.04	0.8	20.03	39.95	0.07
TiNT-side-2	0.25	0.09	42.25	0.03	0.75	18.88	37.65	0.09
Average	0.245	0.0875	40.4925	0.0375	0.8125	19.3675	38.8675	0.08

*Top-1 and 2* top surface of two different implants, *Side-1 and 2* the lateral surface of 2 different implants

Thus, high-resolution Ti2p spectra were obtained to compare the nature of the TiO_2_ oxide layer (Fig. 3c, d). The two dominant peaks in the spectra of the both implant surface types are due to Ti2p1 and Ti2p3 which can be assigned to TiO_2_^26^. The only additional peak visible in the TiMA sample is that for Ti2p3B—the emission due to the underlying Ti metal. The absence of this peak in the TiNT sample shows that the oxide layer is sufficiently thick to prevent electron emission from the underlying metal. Its presence in the TiMA sample indicates that the oxide layer is thin enough to allow electron emission from the underlying metal. Since, in XPS, the penetration depth of electrons is a maximum of 10 nm, it means the oxide layer on the TiMA surface is less than 10 nm but greater than 10 nm on the TiNT surface.

Importantly, while we did not detect any notable chemical differences between the TiMA and TiNT surfaces, differences in their TiO_2_ surface oxide layer thicknesses and their topographical features were obvious.

### µCT imaging of peri-implant bone formation

**Fig. 4:**
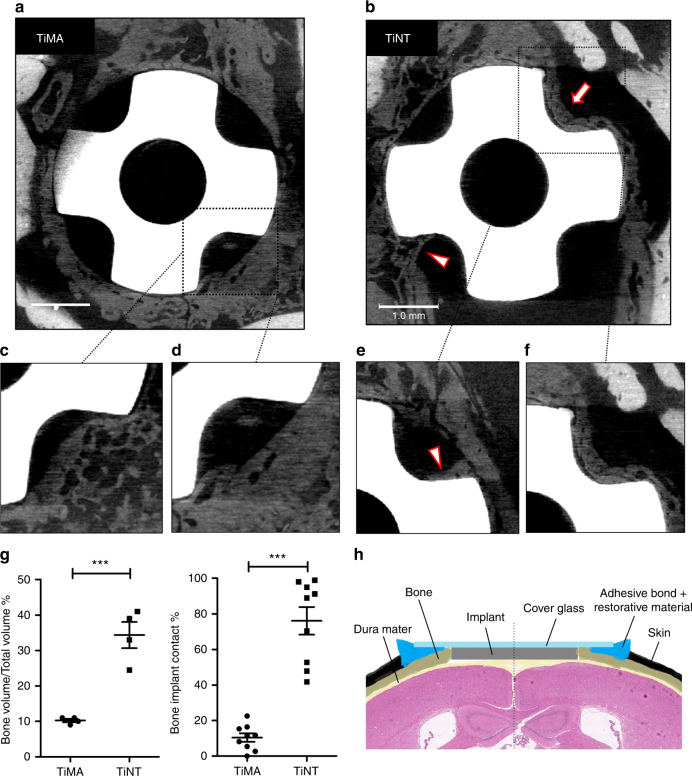
µCT images showing pattern of osteogenesis in response to implant surface topography. Images of the entire calvarial wound site including the Ti implant in the defect with the **a** TiMA and **b** TiNT implants at day 42 post-surgery. Note that the margins of the osteotomy are more easily seen with TiMA implants due to the fact that less bone has grown into the healing volumes. With TiNT implant, in one healing volume the surface of the implant is completely occupied with bone (arrow), and in another bone is growing into the healing volume along the implant surface forming a Baud curve (arrowhead) typical of contact osteogenesis (see text). **c**–**f** Magnified images of the scans in **a**, **b** but at two different depths in the healing volumes around the **c**, **d** TiMA surface and the **e**, **f** TiNT surface. Scale bars (**a**, **b**): 1 mm. **g** Quantitative analysis of bone regeneration parameters (Bone Volume/Total Volume and Bone Implant Contact) in TiMA and TiNT groups at day 42 post-surgery. Data is shown as mean ± SEM (*n* = 4, two-sided Student’s *t*-test performed to compare groups, ***P < 0.001). **h** Schematic showing the coronal view of the CIWC

Samples of the entire skull from both implant groups were scanned at 2, 4, and 6 weeks after implantation using microcomputed tomography (µCT). No bone was detected in the healing volumes at week 2 in the TiNT group. At the end of week 4, contact osteogenesis was observed on the TiNT implant, but not the TiMA group. At week 6 after implantation new bone had been formed in different locations depending on the implant surface topography: on the edge of the craniotomy defect (distance osteogenesis) in TiMA group (Fig. 4a), but directly on the surface of the TiNT implants (Fig. 4b). Individual µCT scans, at two different depths, clearly showed new bone growth into the healing volumes of TiMA was initiated at the craniotomy margin (Fig. 4c, d), while the TiNT samples exhibited osteoconductive bone formation either as a seam of bone on the implant surface or the ingress of bone along the implant surface as characterized by a Baud curve^27^ (Fig. 4e, f and Supplementary Movie 2). Quantitative comparison of the bone volume over total volume (BV/TV%) and bone implant contact (BIC%) showed a significant increase (*P*-value < 0.001) in the amount of bone formed in the healing volume and on the surface of the implant in TiNT samples (Fig. 4g). The schematic in Fig. 4h shows the coronal view of the CIWC in the craniotomy.

### In vivo imaging of neovascularization in the peri-implant wound site

**Fig. 5:**
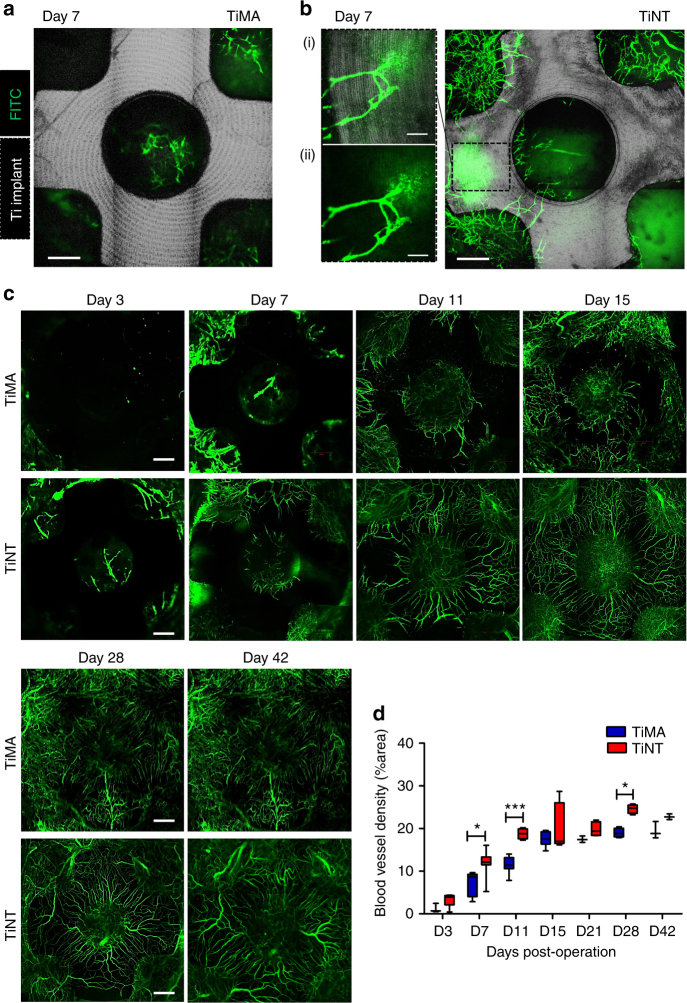
In vivo longitudinal microvascular response to TiMA and TiNT cranial implants. Representative overlaid images of two channels: Silver gray—the reflected light (622–666 nm) Ti implant; Green—the FITC-DEX-labeled vessels at day 7 post-implantation, showing little neovascularization around the **a** TiMA implant compared to **b** TiNT implant. *Notes*: (1) the concentric lines seen clearly on TiMA surface are machining marks and are less obvious on the TiNT sample; (2) the leakage of FITC-DEX from the tip of the incompetent, newly forming, vessels was obvious at higher magnification (**b**.(i)); (**b**.(ii)) Green and silver gray channel overlaid. **c** Representative images demonstrating formation and development of the peri-implant neovascular network from day 3 to day 42 around the TiMA surface and the TiNT surfaced implants. **d** The comparative quantification of functional vessel density (see text) between implant groups over time. *n* = 8 mice/group/time point. Results are mean ± SEM. Two-way ANOVA was performed followed by Bonferroni post-test to compare the mean differences between implant groups over time and at each time point individually. *P-value day 7 = 0.0143, ***P-value day 11 = 0.001, *P-value day 28 = 0.0158. Scale bars (**a**–**c**): 500 µm. **b.**(i), (ii): 200 µm. Images are stacks of tiled scans of the entire craniotomy at the maximum intensity projection; the depth of the field is 0.5 mm, which is equal to the thickness of the implant

The spatio-temporal dynamics of peri-implant wound healing were examined in vivo in C57BL6 mice using our experimental CIWC model. The CIWC remained durable, and infection-free, for up to at least 6 weeks. The CIWC permitted intravital longitudinal tracking of neovascularization at the peri-implant wound site by confocal fluorescence microscopy. Vessels were visualized by tail vein injection of a high molecular weight (2 MDa) fluorescein isothiocyanate-dextran (FITC-DEX) that had a low extravasation rate in intact vessels. Development of the vasculature in the peri-implant healing site was tracked from day 3 to 42 post-implantation. Neovascularization occurred earlier around the TiNT surface than the TiMA surface, and extravasated FITC-DEX was mostly visible from the vessel tips at earlier time points (Fig. 5b.(i), (ii)). Figure 5c shows representative images of vascular development over a period of 42 days around both TiMA and TiNT implants. In the TiMA group, negligible fluorescence signal was detected within the craniotomy defect at day 3. By day 7, some vessels were observed at the periphery of the defect, and within the central implant hole, with evidence of extravasated FITC-DEX. Between days 7 and 11, vessels grew over the top of the TiMA implant surface. Between days 11 and 42, vessels grew in length and while some vessels partially anastomosed, the majority remained fragmented with a disorganized pattern. On the contrary, both the rate and pattern of vascular development around the TiNT implants were different. More vessels had been formed at day 3, and by day 11 the vessels had grown over the top surface of the TiNT implant, anastomosed and formed a dense network. This network was more organized compared to the TiMA group by day 15, exhibiting a less tortuous, predominantly radial and more evenly distributed spatial pattern. Larger vessels were apparent by day 28 and at day 42.

**Fig. 6:**
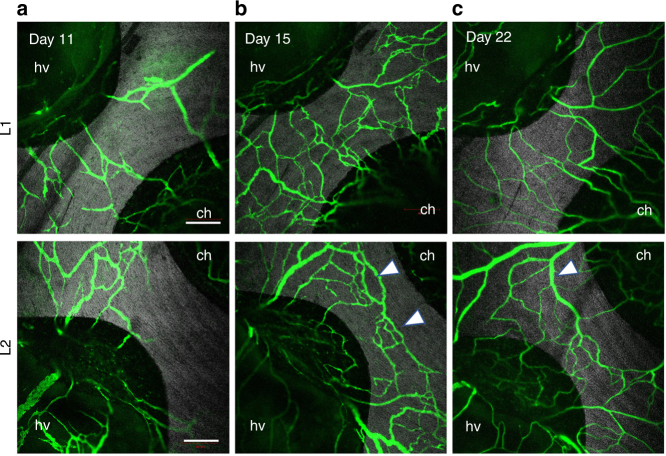
Vascular progression, regression, and remodeling on the TiNT over time. Silver gray—the reflected light Ti implant; Green—FITC-DEX blood vessels at day **a** 11, **b** 15, and **c** 22 post-surgery. Two independent locations (rows L1 and L2) on the TiNT implant surface are shown for each time point. **a** Neovessels formed and partially anastomosed around the Ti implant by day 11. **b** Formation of vascular loops with functional circulation (arrowheads) by day 15. **c** Vasculature remodels to produce fewer but more mature, and larger, functional vessels by day 22. hv healing volume, ch central hole. Scale bar: 500 µm

Comparative analysis of the functional vessel density^28,29^, from weeks 1 to 6, was quantified (as % fluorescent area of each defect) by keeping the concentration and administration dose of FITC-DEX the same in both implant groups, and across all imaging time points (Fig. 5d). Longitudinal fluorescence imaging data showed that the functional vessel density in the TiNT group was higher than the TiMA group at all time points. This difference was significant at the earlier time points, day 7 (*P*-value = 0.0143), day 11 (*P*-value = 0.001), and also at day 28 (*P*-value = 0.0158)—increases of 66.78, 64.5, and 30.1%, respectively. This quantification of the blood vessel density was consistent with the known phases of vascularization—progression, regression, and remodeling—visualized in Fig. 6a–c for two distinct fields-of-view, from days 11, 15, and 22, in TiNT implants.

### The assessment of neovascular morphogenesis

**Fig. 7:**
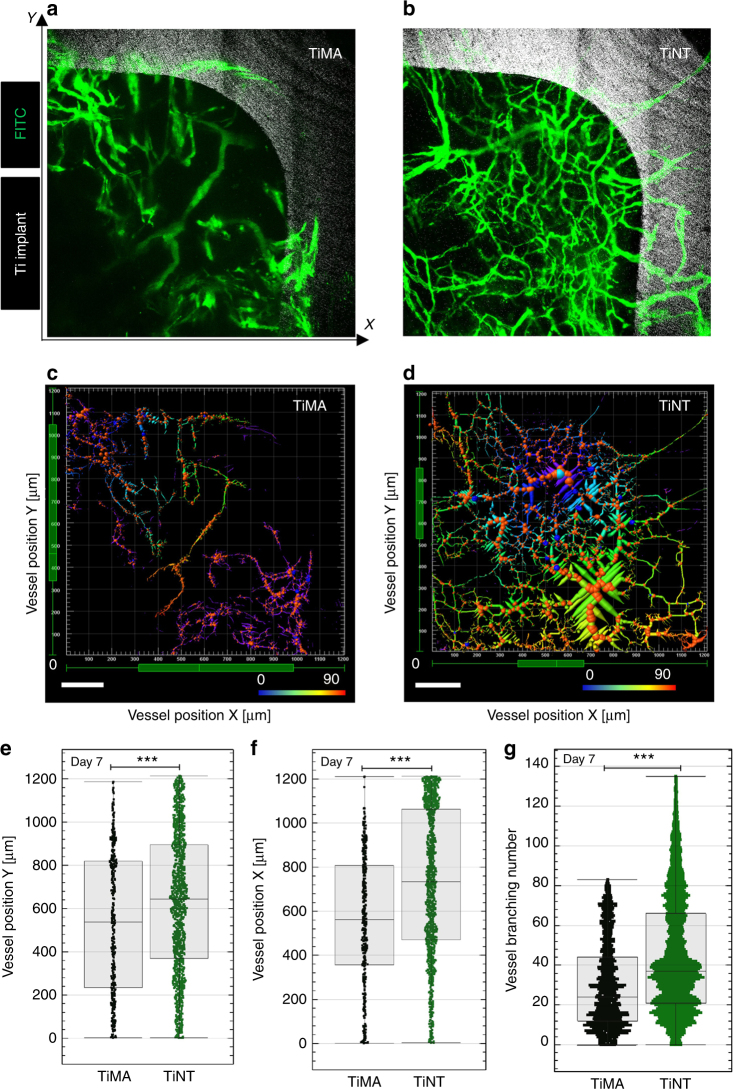
Changes in vascular network structure and branching statistics in response to implant surface topography at day 7 post-surgery. **a**, **b** Images of the vascular network proximal to the TiMA and TiNT surfaces. Green—blood vessels; Silver gray—Ti implant. Each healing volume can be described by three Cartesian coordinates. The *X* and *Y* axes are marked (the *z*-axis would be the depth of the healing volume). **c**, **d** Corresponding 3D image skeletons showing the spatial distribution of the vessel segments in two representative HVs. The coordinates were anchored by defining the bottom left corner of the HV as the point of reference (0), and the implant was located at the top right of the HV. The color coding represents the vessel branching number. Scale bar is 200 µm. **e**, **f** Box plots represent the quartile distribution of the vessel segment coordinates along the *X* and *Y*-axis in TiMA (Black) and TiNT (Green) groups, with the whiskers representing the 0 reference point and the surface of the implant, respectively, on the *X* and *Y* axes. The individual data points are superimposed along the whiskers as scatter plots. The higher the value *X* or *Y*, the closer the segments were to the lateral surface of the implant. **g** Comparison of the branching number between TiMA and TiNT groups. *n* = 8 animals/group/time point. Error bar = median ± IQR. Mann–Whitney test was used to assess the statistical significance of the two medians ***P-value < 0.001

To characterize the morphology of the vasculature developed proximal to the implants at early time points after implantation, morphometric analysis was performed on the confocal intravital images of the FITC-DEX taken at days 7 and 15 post implant surgery. An example of a healing volume from each of the TiMA and TiNT groups, respectively, is shown in Fig. 7a and b. The greater degree of neovascularization in the TiNT group is evident, while the less well-developed vessels around the TiMA group show a more leaky appearance. Figure 7c and d shows the vascular skeletons corresponding to Fig. 7a and b, which were used to identify the vessel segment coordinates and measure the following vascular morphometric parameters: vessel branching number, vessel volume, and vessel length. The vessel density and vessel length are a measure of the quantity and the continuity of the vessels, respectively. The vessel volume is a 3D fluorescence-based measurement of the entire vascular volume in the peri-implant wound site occupied by intravascular FITC-DEX, which provides an estimate of the total blood volume in the wound site. According to the box plots in Fig. 7e and f, the TiNT vessel segment coordinates were significantly higher (*P*-value < 0.0001) than those of the TiMA implants in both axes (25% and 30% increase in the median values for *X* and *Y* coordinates, respectively). The number of vessels within the top quarter percentile is higher in TiNT compared to TiMA implants as can be seen from both the range and number of data points within the top quarter percentile. As the top quarter percentile is the closest spatial region to the implant lateral surface, these data show that the number of vessels in proximity to the TiNT surface was significantly higher than seen with the TiMA surface (*P* < 0.0001 for both *X* and *Y* coordinates).

**Fig. 8:**
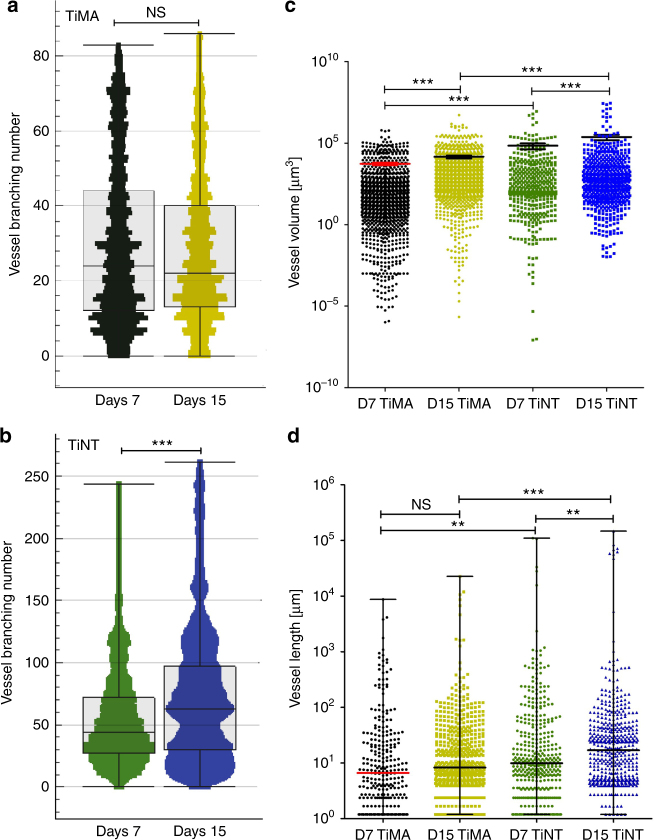
Comparison of the vascular morphometric parameters between implant types characterizing the features of the vessel network: branching number; vascular volume, and vessel length from day 7 to day 15 post-surgery. In all: Black = TiMA day 7; Gold = TiMA day 15; Green = TiNT day 7; Azure = TiNT day 15. Branching number of the network in **a** TiMA and **b** TiNT groups. Error bar = median ± IQR. *n* = 8 animals/group/time point. Mann–Whitney test was used to assess the statistical significance of the two medians ***P-value < 0.001. **c** comparison of the vessel volume in both TiMA and TiNT implant groups in day 7 and 15 post-surgery; error bar = mean ± SEM. **d** comparison of the vessel length in both TiMA and TiNT implant groups in day 7 and 15 post-surgery; error bar = median ± IQR. In **c**, **d**, *n* = 8 animals/group/time point. Kruskal–Wallis test was performed followed by a Dunn’s multiple comparison test. NS not significant, **P-value < 0.01, ***P-value < 0.001

At day 7, the TiNT group exhibited a hierarchically branched network of the vessels with small branches that grew over the surface of the implant and distributed along the lateral surface (Fig. 7d). The vessel branching number, which is a measure of vessel sprouting in a developing microvascular network, was significantly higher in the TiNT (136) than the TiMA group (83), at day 7 (Fig. 7g) (*P*-value < 0.001). The vessel branching number did not change from day 7 to day 15 in the TiMA group (Fig. 8a), while there was a 92.5% increase of the maximum in the TiNT group, with a median increase of 89% (Fig. 8b). Assessment of the vascular network volume, which represents the volume of the blood flow within the peri-implant wound site, showed a significant increase in both implant groups between weeks 1 and 2 (Fig. 8c). However, at both days 7 and 15, the mean vascular volume was significantly higher around the TiNT surface (69,858 and 240,440 µm^3^, respectively) compared with the TiMA surface (5422 and 14,575 µm^3^, respectively). A similar trend was observed in vessel length data (Fig. 8d), with no significant difference between weeks 1 and 2 in the TiMA group. The distribution of the vessel length around the TiNT implant at day 7 was similar to day 15, ranging from very short branches to long branches. However, the fold increase (106%) in median length sum by day 15 in the TiNT group is suggestive that the shorter branches have been remodeled to form longer branches.

## Discussion

While neovascularization is an essential prerequisite to osteogenesis, no previous published reports have examined the effect of implant surface topography on the spatio-temporal pattern of neovascularization during endosseous peri-implant healing in vivo. Our results clearly show that the surface design of the implant has a profound effect on the pattern of neovascularization with new vessels being developed at, or near, the implant surface and the vascular network maturing through remodeling sooner in the presence of a topographically complex surface. The rapid development of a functional vascular supply is of key importance to peri-implant wound healing, both as a source of scavenging and immune-modulating leukocytes, and a nutrient supply to support tissue regeneration. Indeed, the rate of osseointegration is critically dependent upon osteoconduction—the key determinant of contact osteogenesis^30^—and we have shown, quantitatively, that this can be accelerated by increasing the topographic complexity of the implant surface^31^. Thus, our findings provide a new perspective on the importance of implant surface design that is relevant to many therapeutic areas, including orthopedics, dentistry, otorhinolaryngology, and plastic surgery. Previous studies have established the window chamber model as a tool to longitudinally image the spatio-temporal development of both neovascularization and osteogenesis in craniotomies^32,33^. An observation common to these, and microCT, calvarial studies is that new bone grows centripetally within the bony defect both in the unmodified state^34,35^ or when the defect is modified by the addition of growth factors^34,35^, cells^36^, or cells and scaffolds^36–38^. This is important because we show, on the contrary, that when a metallic implant is introduced into such a model, the pattern of bone growth is modulated as a function of implant surface topography: as expected the TiMA (smoother) and TiNT (rougher) surfaces exhibited distance and contact osteogenesis, respectively^1^. This observation provides an essential validation of our CIWC model as it has been generally accepted, since the work of Buser et al.^12^, that implant surface topography has a profound effect on contact osteogenesis. Indeed, we have established the functional significance of three distinct scale ranges of implant topography on both bone bonding and bone anchorage, two distinct mechanisms within the phenomenon of osseointegration^39^. It is worth mentioning that in the current study we have only investigated the effect of implant surface topography on peri-implant neovascularization using two surfaces, a relatively smooth machined (TiMA) surface and a complex microtopographic surface with superimposed nanotubes (TiNT). However, we believe our platform would be suitable for studying spatio-temporal vascular morphogenesis around other surfaces beyond those discussed in the present paper.

Our model has enabled direct visualization of three distinct phases of vascularization during the first 42 days of healing: capillaries sprouted and grew longer, anastomosed to form loops, and, finally, remodeled into a more functional vasculature that facilitated blood flow throughout the peri-implant site. High-resolution images showed that the vasculature grew predominantly from the periphery of the bony defect toward the lateral surface of the implant, but vessels also grew from the dural surface into the central implant hole. With time this peripheral and central vasculature anastomosed on the top flat surface of the implant, with blood flow in each direction (Supplementary Movie 1). Although such anastomoses occurred on both the machined and topographically complex surfaced implants, only the latter displayed an ordered, radial, arrangement of vessels, a pattern completely absent on the machined surface, during the time course of our experiments. Indeed, we demonstrated that the TiNT surface not only increased the rate of neovascularization following endosseous implantation, but also changed the morphological characteristics, spatial pattern, and functionality of the re-established microvasculature. Interestingly, while the machining marks were obvious on the machined implant, they were less evident on the complex surface. There have been numerous reports of cell migration along the long axes of surfaces with linear features^40,41^, but we saw no evidence that these topographic features influenced the directional growth of vessels.

At the earliest days of healing, in both implant groups, the neovessels were highly permeable as FITC-DEX extravasated from both the lumen and ends of the nascent vessels, appearing as a bloom of fluorescence. With time, and increasing function, extravasation of the FITC-DEX through the vessel walls was reduced and only leaked out from the vessel tips. We believe that such extravasation is due to the immaturity of the distal blood vessels, since it was absent at later time points.

Morphological properties of the microvascular system affect the blood flow and its distribution within the wound area^42^. The morphometric parameters used in this study which were measures of vascular density, volume, length, and branching number are indicators of the ability of the vasculature to distribute flow throughout the tissue. These are standard parameters used by several studies assessing physiological^32,43^ or pathological angiogenesis^44,45^, although representation of the data on combined box-and-whisker and scatter plots provides additional graphic information concerning the frequency distribution of the individual vessels in the complex 3D network.

From the physiological standpoint, distribution and collection of blood-borne substances within tissues and organs requires a branching system. Hierarchical branching of a vascular network—starting from a relatively large stem vessel to smaller and smaller branches—is essential for conducting flow further into the wounded area. However, a non-optimal vascular density reduces vascular functionality^46^. Therefore, the pruning of excessive vessels is essential for maturation of a vascular network. The branching number shows increased branching around TiNT implants compared to TiMA implants. The early dense network of small vessels matures, through remodeling, to larger functional vessels that conduct a higher volume of the blood. During the maturation of the vascular network some of the morphological features such as vessel length, volume, and branching change concomitantly as there are scaling relations between these parameters^47^. The choice of one vessel over another in the pruning process is known to be based on blood flow^48^. Vessels with higher blood flow increase in girth while those with lesser blood flow regress. Our results show a higher mean vessel volume in the TiNT group both at weeks 1 and 2 compared to the TiMA group. However, the number of vessels is higher in the TiMA group. This indicates that large vessels have an essential role in increasing the bulk flow compared to numerous small vessels. By week 4, the vascular network was remodeled to form larger vessels that improved functional blood flow for both implant types. This measure of blood flow is important since it has been reported that the progenitor cells position themselves relative to the volume of the blood^49^, and vessels were consistently larger around the TiNT implants.

Since we would not expect to image vessels that may have formed independent of the pre-existing vascular network, as they would not be labeled with FITC-DEX unless they had anastomosed with those that had developed from the functional vasculature of the circulation, we cannot exclude the possibility of vasculogenesis as distinct from angiogenesis^50^. However, our results do show that the changing characteristics, structural organization, and spatial location of the re-established vascular network around the two implant surfaces was reflected in a corresponding change in the spatial pattern of bone healing. Previous cranial defect healing models have suggested that the osteogenic precursor cells can originate from the periosteum, bone marrow (BM)^51,52^, and dura matter^53^, and we would expect these tissue-resident mesenchymal cells to be of perivascular origin^54^, although not pericytes^55^. In fact, Hung et al. showed that there is a correlation between the morphometric characteristics of the vascular network, particularly the diameter and the length of the blood vessels and the volume of the differentiated osteoblasts in their vicinity^32^. Thus, by altering the surface characteristics of the implant, which we have shown to have profound effects on neo-vascularization, the ingress of osteogenic precursors, and their location with respect to the implant surface is also being affected, resulting in either contact or distance osteogenesis.

In contradistinction to previous reports, our model provides a unique and reproducible preclinical platform to study implant healing biology over clinically relevant time scales. The window is durable for more than 6 weeks, sufficient to monitor early critical stages of both peri-implant neovascularization and osteogenesis. Using intravital imaging, we obtained both qualitative and quantitative information on the complex 3D structure of the neovascularization with respect to the two different implant surfaces over a large region of interest (ROI) (4 mm). Tracking active vascularization from initiation to remodeling in a single animal, over multiple time points, reduces animal-to-animal variation and increases the reliability of the quantification. Interestingly, the presence of the implant blocked much tissue auto-fluorescence and resulted in an increased signal-to-noise ratio. Together with longer pixel dwell, these details may account for the higher-resolution images we obtained compared to previous intravital studies^32,33^. Ti-based implant materials are commonly employed in orthopedic, craniofacial, and dental surgery due to their combination of mechanical properties, corrosion resistance, and biocompatibility^56–58^. Our results show that a topographically complex surface contributes to the development of a radially arranged vascular structure with hierarchical branches spatially closer to the surface of the Ti-implant. These findings emphasize the translational importance of a rationale for implant surface design, which could help improve the clinical effectiveness of endosseous implants compared to traditional implant surfaces.

As neovascularization is the route for the ingress of both immune and progenitor cells, alterations in the surface topography would enable healing through regulation of neovascularization. Intravital studies could throw more light on the cellular and molecular mechanisms by which implant surface topography affects both vascularization and osseointegration. Furthermore, the recent development of a femur window chamber model^59^ may allow more precise mimicking of the cortico-cancellous bone environment in which dental and orthopedic implants are normally placed. A comprehensive understanding of the healing and regeneration mechanisms of endosseous integration in the peri-implant niche could have a considerable impact in implant medicine. The knowledge transferred from the current study provides one step forward toward designing endosseous implants capable of controlling endogenous peri-implant vascularization.

## Methods

### Animal studies

All animal procedures conducted in accordance to institutional animal use guidelines approved by University Health Network animal care committee (AUP #4884.0-1). Nine to eleven-week-old male C57BL6 mice (Charles River Laboratories, Quebec) were used for the entire study.

### Ti cranial implants

The implants were custom-made from grade IV commercially pure Ti, specifically for this study, by ZimmerBiomet Dental (Palm Beach Gardens, FL). The implants were machined from a 4 mm rod stock with a central 2 mm drill hole. Four radially equidistant flutes, with internal radii of 0.5 mm, were machined along the length of the rod. The rod was then machine-sliced, resulting in flat, 4 mm diameter and 500 µm thick, implant forms with the cruciate shape as seen in Fig. 1a. The topographies of all surfaces of such implants bore the marks of the machining process. The first cohort of the machined implants were left unmodified (TiMA) while the second cohort (TiNT) was further modified by bolting multiple implants together, through the central hole, and using guide bars to ensure that the flutes were aligned longitudinally. The outer surfaces were then grit blasted [325–450 µm particle size range] dual acid etched in 8% hydrofluoric acid (HF) followed by 78% H_2_SO_4_/3% HCl (vol%), and TiO_2_ nanotubes (TiNT) were created on this modified surface by electrochemical anodization. For this, the machined implants were ultrasonically cleaned in concentrated detergent followed by rinsing in deionized (DI) water. The TiNT implants were anodized in an electrolyte consisting of 0.250 wt% HF (Sigma-Aldrich). The Ti implant served as the anode while a cp-Ti electrode served as the cathode. Both were connected to a power supply (BK Precision 9602) at 20 V and immersed in the electrolyte solution with stirring at room temperature for 30 min. After anodization, the implant was rinsed with DI water and air dried at 120 °C for 1 h in a forced convection oven. The central bolt was removed and the modified individual implants were ultrasonically cleaned in acetone, 70% ethanol, and DI water, and subsequently autoclaved at 121 °C for 20 min. A total of 10 TiMA and 10 TiNT implants were used in this study. The resulting implants, therefore, had a cruciate form of 4 mm external diameter, a central hole of 2 mm, and four cut-outs that provided four separate tissue healing volumes. Implants were individually packaged and sterilized by gamma irradiation.

### Implant surface characterization

*Field-emission scanning electron microscopy*. Two Ti implants from each surface group were removed from the sterile packs with plastic tweezers and fixed with carbon tape to SEM stubs, taking care to not to damage or contaminate the surfaces. Both the lateral and flat surfaces of the implants were imaged non-coated at an accelerating voltage of 5 keV and increasing magnifications (up to 50,000×) by FE-SEM (Hitachi S-5200, Japan).

*X-ray photoelectron spectroscopy*. Implants were analyzed by XPS using a Thermo Fisher Scientific K_α_ spectrometer (E. Grinstead, UK). A monochromatic Al K_α_ X-ray source was used with a nominal 400 µm spot size. Survey spectra were obtained (200 eV pass energy (PE)) followed by an examination at 150 eV PE of spectral regions of interest from which the relative atomic percentage composition was obtained. High-resolution spectra (25 eV PE) were also obtained for the Ti envelope. Charge compensation was applied for all spectra using a combined e/Ar + floodgun, and the energy scale was shifted to place the C1s peak at 284.6 eV. All data processing was performed using the Avantage 5.926 software supplied by the manufacturer.

### Surgical procedure for CIWC placement

The surgical procedures were performed in a microsurgery room under aseptic conditions on a microsurgical table. Mice were anesthetized with Isoflurane 2.5% vaporized in a 70/30 mixture of O_2_/N_2_O. The scalp was shaved and the skin was cleaned with Betadine solution and 70% ethanol. The skin was lifted with tweezers from the midpoint of the ears, cut with fine curved dissecting scissors, and completely removed to expose an 8 mm diameter circular area in the underlying skull. The periosteum was reflected using a periosteal elevator. Once the calvaria was exposed, a midline 4 mm diameter osteotomy was carefully created in the parietal bones using a custom-made trephine (ZimmerBiomet Dental, FL) under continuous irrigation with sterile saline. A guidance stop-line, laser-marked at 200 µm from the tip of the trephine, minimized over-penetration into the craniotomy site during the surgery. The created circular bone piece within the osteotomy was elevated using a periosteal elevator, taking care to leave the dura mater intact. An implant was then placed into the osteotomy. A permanent intracranial imaging window was superimposed over the implantation site to permit imaging through the depth of the healing volumes (and central implant hole), secure the implant, and inhibit the growth of the skin over the defect (Fig. 1b).

To fix the imaging window, the exposed skull around the osteotomy was first covered with Scotchbond dual cure adhesive resin and then a ring of dental restorative material (3 M, Milton, ON) was applied on top of the bonding agent but maintaining a 3 mm distance from the edge of the craniotomy. A round coverslip, 8 mm in diameter, #1 thickness (neuVitro, Germany) was positioned on top of the restorative ring, pressing down gently to secure the Ti implant in the craniotomy. The restorative material was then light-cured to ensure a perfect seal around the defect and to stabilize the coverslip on top of the defect. Physiological body temperature was maintained throughout the surgery and recovery time by a homeothermic pad and healing lamp. Animals were carefully monitored after CIWC placement and they resumed normal activities within 3 days.

### Intravital confocal laser scanning microscopy

Intravital imaging was performed post-operatively to track the microvascular changes during peri-implant healing. Prior to each imaging session, mice were anesthetized by standard intraperitoneal injection of a ketamine/xylazine mixture (80/13 mg/kg). Each mouse was then administered FITC-DEX (2 MDa; 0.1 mg/mouse; 200 µL injected/mouse) via the tail vein using an ultra-fine 6 mm insulin syringe.

Creation of motion artifacts caused by respiration was controlled and minimized by stabilizing the mouse head on modeling clay and resting the body on a heated stage. In addition, the imaging window was fixed in place by fitting it into a metallic restrainer as demonstrated in Fig. 1c and d. The imaging procedure was followed according to the experimental timeline shown in Fig. 1e. The confocal/two-photon fluorescence imaging was performed using LSM 710 (Carl Zeiss, Germany). Using the *XYZ*-axis controller, the crucial landmark locations, such as bone–implant interface, were identified through the CIWC. Once the implant interface and the adjacent bone were found, the images were acquired using 5×, 10×, and 20× water immersion objectives. Images were acquired with 488 nm excitation and 500–550 nm emission at 1024 × 1024 pixels and 0.79 μs pixel dwell. The implant was visualized by collecting reflected light in a second channel (633 nm excitation, 622–666 nm emission).

To obtain 3D images of the CIWC, the points above and below the implant in the *z*-plane were defined by driving the microscope to a point just out of focus on both the top and bottom of the implant surface. Images were recorded as a series of TIF files with dimensions of 1024 × 1024 pixels. Stacks of images were collected for the FITC channel with Z-stack size ≃500 μm. Image acquisition settings were maintained consistent throughout all time points and groups.

### Image processing and analysis

Fluorescent images were processed in Zen lite (Zeiss, Jena, Germany) and ImageJ. A MATLAB-based computational code was developed to calculate the functional vessel density. To calculate functional vessel density, maximum intensity projection images of the z-stacks were obtained, binarized, and the positive pixel percentage area was calculated for each ROI.

Quantitative spatial analysis of the vascular network structure in 3D was performed using the Imaris (ver. 8.3.0, Bitplane, AG, Switzerland). The four healing volumes represented four ROIs that we identified and analyzed at each imaging time point. To measure vessel parameters, each implant healing volume is first oriented in the same manner, as shown in Fig. 7a and b. Each healing volume can then be described by three Cartesian coordinates, the *X* and *Y* axes are marked in Fig. 7b (the *z*-axis would be the depth of the healing volume, which is not seen in this plan projection). Stacks of images were analyzed using the filament tracer function. The size of each ROI was 1200 × 1200 mm^2^, and all four ROIs were set at the same orientation to maintain the coordinates consistent across all images. A semi-automatic looping algorithm was used to detect and skeletonize the vascular network. The following definitions were employed: filament: the stem vessel including all branches; segment/branch; the distance between two branch points or between a branch point and a beginning/terminal point in a filament. Filaments are the building blocks of a vascular network. The following parameters were analyzed: vessel branching number; the number of branch points in the shortest path from the beginning point to a given point in a filament. Vessel position *X* and *Y*: the *X* and *Y* coordinates of a vessel segment positioned on an XY plane with respect to a reference point. Vessel volume: the sum of the volumes of all segments within the entire filament, and vessel length: the sum of the length of all segments within the entire filament.

### Sample harvesting and ex vivo micro-CT imaging

The animals were euthanized by exposure to CO_2_ at days 14, 28, or 43 post-surgery. The complete skull was harvested and fixed in 10% formalin for at least 48 h. Following fixation, the mandible and the brain were removed, and the dura was kept intact. The samples were further trimmed to remove excess tissue for µCT scanning.

Prepared trimmed samples were scanned using a MicroCT40 (Scanco Medical, Switzerland) at 70 kVp and 114 µA. Images were acquired with a high resolution in three planes, creating slices of 6 µm thick. A ROI that included the entire defect area was selected, and highlighted in the cross-sectional images from each specimen. ROIs were then reconstructed in 2D enabling visualization of bone formation in each of the four healing volumes in each implant. 2D images were used as a qualitative demonstration of the mechanism of bone formation (contact vs. distance osteogenesis) at the healing volumes.

### Statistical analysis

Temporal series results (Days 3–28) were presented as mean ± SEM, and analyzed by two-way repeated measures analysis of variance (ANOVA) in Graphpad. Bonferroni post-tests were performed to test the significance of the means between implant groups at each time point. A confidence level of 95% was considered significant. The in vivo optical imaging procedure was repeated with 6–8 animals per time point per implant group. To obtain the statistics of the (vessel) filaments, a D’Agostino-Pearson normality test was performed to assess the normality of all data sets. As the data were not normally distributed, where two implant types were compared at one time point, Mann–Whitney test was used to assess the statistical significance of the two medians; Interquartile range (IQR) has been shown on the scatter dot Mann–Whitney plots. Where comparing three or more groups of data, a Kruskal–Wallis test was performed followed by a Dunn’s multiple comparison test. *P*-values < 0.01 were considered significant.

### Data availability

The MATLAB-based computational code for calculation of the functional vessel density has been deposited in figshare with the identifier DOI 10.6084/m9.figshare.6201062.v1. Supporting images are available from the authors upon request.

## Electronic supplementary material

Description of Additional Supplementary Files

Supplementary Movie 1

Supplementary Movie 2
