# Supplementary material for: Nanosurfaces modulate the mechanism of peri-implant endosseous healing by regulating neovascular morphogenesis
Source: Commun Biol. 2018 Jun 18;1:72. doi: 10.1038/s42003-018-0074-y (PMC6123776; doi:10.1038/s42003-018-0074-y)
Supplement: Supplementary file 1 — Description of Additional Supplementary Files [file 42003_2018_74_MOESM1_ESM.docx]

**Description of Additional Supplementary Files**

File Name: Supplementary Movie 1

Description: **Intravital live video of peri-implant neovasculature**. Green—the FITC-DEX labeled vasculature, video has been taken by a confocal fluorescence microscope through the cranial implant window chamber implanted in the calvaria of a 11-weeks-old mouse. Peripheral and central vasculature anastomosed on the top flat surface of the implant, facilitating the blood flow in each direction.

File Name: Supplementary Movie 2

Description**: µCT images of the entire healing volume around a Titanium implant which is nanotopographically modified (TiNT) at day 42 post-surgery**. The stack of the images along the Z axis shows different slices from the top (cover glass as seen in the ground glass appearance due to shadowing) through the depth of the healing volume. Formation of the bone on the surface of the implant is apparent. As the endocranial periosteum (dura mater) is approached, we see no signs of bone formation originating from the dural surface.
